# Hospital-wide cardiac arrest in situ simulation to identify and mitigate latent safety threats

**DOI:** 10.1186/s41077-022-00209-0

**Published:** 2022-05-21

**Authors:** Suzanne K. Bentley, Alexander Meshel, Lorraine Boehm, Barbara Dilos, Mamie McIndoe, Rachel Carroll-Bennett, Alfredo J. Astua, Lillian Wong, Colleen Smith, Laura Iavicoli, Julia LaMonica, Tania Lopez, Jose Quitain, Guirlene Dube, Alex F. Manini, Joseph Halbach, Michael Meguerdichian, Komal Bajaj

**Affiliations:** 1 Simulation Center at Elmhurst, NYC Health + Hospital/Elmhurst, Elmhurst, NY USA; 2 Department of Emergency Medicine, Icahn School of Medicine at Mount Sinai, New York, NY 10029 USA; 3 Department of Medical Education, Icahn School of Medicine at Mount Sinai, New York, NY USA; 4 NYC Health + Hospitals/Elmhurst, Elmhurst, NY USA; 5 Department of Anesthesiology, NYC Health + Hospitals/Elmhurst, Elmhurst, NY USA; 6 Patient Experience, NYC Health + Hospital/Elmhurst, Elmhurst, NY USA; 7 Department of Obstetrics and Gynecology, NYC Health + Hospital/Elmhurst, Elmhurst, NY USA; 8 Department of Obstetrics and Gynecology, Icahn School of Medicine at Mount Sinai, New York, NY USA; 9 Pulmonary and Critical Care, NYC Health + Hospital/Elmhurst, Elmhurst, NY USA; 10 Emergency Medicine, NYC Health + Hospitals/Elmhurst, Elmhurst, NY USA; 11 Pediatrics, NYC Health + Hospitals/Elmhurst, Elmhurst, NY USA; 12 Division of Medical Toxicology, Department of Emergency Medicine, Icahn School of Medicine at Mount Sinai, New York, NY USA; 13 Department of Emergency Medicine, NYC Health + Hospitals/Harlem, New York, NY USA; 14 Simulation Center of NYC Health + Hospitals, New York, NY USA; 15 NYC Health + Hospital/Jacobi, Bronx, NY USA; 16 Department of Obstetrics & Gynecology, Albert Einstein College of Medicine, New York, NY USA

**Keywords:** Patient safety, Quality, Simulation, Cardiac arrest, Latent safety threat

## Abstract

**Background:**

Cardiac arrest resuscitation requires well-executed teamwork to produce optimal outcomes. Frequency of cardiac arrest events differs by hospital location, which presents unique challenges in care due to variations in responding team composition and comfort levels and familiarity with obtaining and utilizing arrest equipment. The objective of this initiative is to utilize unannounced, in situ, cardiac arrest simulations hospital wide to educate, evaluate, and maximize cardiac arrest teams outside the traditional simulation lab by systematically assessing and capturing areas of opportunity for improvement, latent safety threats (LSTs), and key challenges by hospital location.

**Methods:**

Unannounced in situ simulations were performed at a city hospital with multidisciplinary cardiac arrest teams responding to a presumed real cardiac arrest. Participants and facilitators identified LSTs during standardized postsimulation debriefings that were classified into equipment, medication, resource/system, or technical skill categories. A hazard matrix was used by multiplying occurrence frequency of LST in simulation and real clinical events (based on expert opinion) and severity of the LST based on agreement between two evaluators.

**Results:**

Seventy-four in situ cardiac arrest simulations were conducted hospital wide. Hundreds of safety threats were identified, analyzed, and categorized yielding 106 unique latent safety threats: 21 in the equipment category, 8 in the medication category, 41 in the resource/system category, and 36 in the technical skill category. The team worked to mitigate all LSTs with priority mitigation to imminent risk level threats, then high risk threats, followed by non-imminent risk LSTs. Four LSTs were deemed imminent, requiring immediate remediation post debriefing. Fifteen LSTs had a hazard ratio greater than 8 which were deemed high risk for remediation. Depending on the category of threat, a combination of mitigating steps including the immediate fixing of an identified problem, leadership escalation, and programmatic intervention recommendations occurred resulting in mitigation of all identified threats.

**Conclusions:**

Hospital-wide in situ cardiac arrest team simulation offers an effective way to both identify and mitigate LSTs. Safety during cardiac arrest care is improved through the use of a system in which LSTs are escalated urgently, mitigated, and conveyed back to participants to provide closed loop debriefing. Lastly, this hospital-wide, multidisciplinary initiative additionally served as an educational needs assessment allowing for informed, iterative education and systems improvement initiatives targeted to areas of LSTs and areas of opportunity.

**Supplementary Information:**

The online version contains supplementary material available at 10.1186/s41077-022-00209-0.

## Background

Cardiac arrest resuscitation requires optimized teamwork and communication for safe and effective care. There are an estimated 292,000 annual in-hospital cardiac arrests in the USA but only a 25.8% survival rate [1]. The team responsible for cardiac arrest victims varies depending on the location within a hospital, as well as the respective hospital policy; however, cardiac arrest victims are consistently cared for by interdisciplinary teams trained in advanced cardiac life support (ACLS) and/or pediatric advanced life support (PALS) [2]. This study focuses on the key event of a cardiac arrest. Because cardiac arrest frequency greatly varies based on the unit, often times, a threat cannot be appreciated until after an event. This unique study in which cardiac arrest simulations were run hospital wide with the real cardiac arrest clinical teams in the actual clinical arena as if a real event allows for crucial systems’ evaluation and key insights into team dynamics and vulnerabilities in the system. Furthermore, performing these simulations unannounced to participants maximized realism allowing for a more complete and accurate capture of threats to patient safety.

Knowledge and clinical experience alone do not translate to effective teamwork without deliberate practice and team training, both of which can be achieved through simulation [3]. Simulation offers opportunities for deliberate practice, adjustable clinical complexity, and regular execution of rare or complex events such as cardiac arrests [3]. Previous studies show the importance of simulation and training in teamwork dynamics (e.g., communication and leadership) on team performance, nontechnical skills, communication, and clinical outcomes [4–9]. For example, a trauma team simulation initiative demonstrated improved time-to-task completion, increased task completion, and increased teamwork scores, both during simulations, and sustained in observed real-life traumas following the simulation initiative [10]. The TRUST study, a trauma simulation initiative, revealed over 150 critical latent safety threats (LSTs) in 12 simulations with criticality determined by expert scoring based on perceived frequency and severity of threats [11].

Another powerful tool for teamwork training and team and system assessment is in situ simulation. In situ simulation can be thought of as “crash testing the dummy” [12] or more formally can be defined as taking place in the actual patient care setting/environment in an effort to achieve a high level of realism using authentic equipment, resources, and healthcare team members from that unit [13]. This allows for simulation to be used not only as an educational modality but also to “test” the system and uncover issues. LSTs are defined as “system-based threats to patient safety that can materialize at any time” and often go unrecognized [14]. Reason’s Swiss cheese model is one example of a systems perspective of error causation in which he describes not just one factor leading to an error or negative outcome but rather, like swiss cheese, many holes or latent threats that must all align for the active error to materialize [15].

Active errors are attributed to the immediate action of a human to cause an error, while LSTs allow active errors to cause an adverse event or harm the patient [15]. Addressing an active error may provide only a temporary fix if one does not also address the surrounding latent conditions that allowed the error to have an impact. LSTs are essentially “errors waiting to happen,” and enhancing safety requires focus on preventing errors and identifying and improving latent conditions before active errors occur [14–16].

Using in situ simulation with immediate team debriefing, LSTs may be identified in the real clinical environment by evaluating the actual conditions under which individuals work and strategically working to build defenses to remove, minimize, or mitigate errors [15, 17]. Experience alone may not lead to learning without facilitated and deliberate reflection on the experience, as afforded via debriefing [18]. Whether in a simulation center or in situ, a simulated experience maximizes the opportunity for immediate debriefing with greater facilitator control of rarer events, a reflective conversation that often does not occur as regularly in clinical practice after real cases [18–23]. Through in situ simulation with the entire multidisciplinary team, LSTs can not only be identified by the observers and facilitators but most importantly by the participants in the simulation itself. These team members are the most familiar with their own unit and resources and can identify systems’ issues that affected performance in the simulation [24]. In situ conditions, in this regard, potentially allow for a better evaluation of LSTs as the system can be tested where patient care actually happens.

### Objectives

Cardiac arrests pose major theoretical patient safety threats due to their high-stakes nature, variable frequency across hospital locations, and unique challenges in care due to variations such as responding team composition, time since the last cardiac arrest event, and familiarity with arrest equipment. This initiative endeavors to utilize these simulations and accompanying debriefings to systematically assess, capture, and analyze areas of opportunity and LSTs hospital wide, as well as key challenges unique to specific locations, in order to mitigate threats and maximize patient safety.

## Methods

### Setting

This single-center quality improvement initiative was conducted hospital wide in multiple different clinical areas in order to evaluate cardiac arrest management (Table 1). Team functioning and the composition of responding cardiac arrest team members vary depending on location, provider availability and proximity to the event, and other variables, such as time of day or night. These variations make hospital-wide in situ simulation crucial to assess the overall functioning of cardiac arrest teams and work to expand the safety net around these high-stake events. In the ED and medical intensive care unit (ICU), cardiac arrest team members are comprised internally of healthcare providers and staff from the specific area (without overhead or pager activation for additional response). In comparison, inpatient adult floors and the majority of remaining hospital areas are covered by the hospital cardiac arrest team comprised of providers from across the hospital that join the local providers and staff in the patient location at varied arrival times. When a patient suffers a cardiac arrest, the cardiac arrest team is activated through a pager system coupled with overhead announcement of location. The multidisciplinary team responding from the hospital includes a senior internal medical resident (responsible for leading the cardiac arrest team), additional internal medicine residents, anesthesiologist(s), respiratory therapist(s), a patient transporter (to deliver an additional crash cart), a nurse manager carrying the cardiac arrest team pager, among others. In the interim prior to arrival of the cardiac arrest team, ACLS, PALS, or basic life support (BLS) are initiated and followed by the healthcare staff in the respective unit within their respective scopes of practice and to the best of their abilities. Additional differences apply based on location, such as labor and delivery, where in addition to activation of the cardiac arrest team, there is a notification to pediatrics/neonatology if a gravid mother.

**Table 1:** Cardiac arrest scenario by hospital location and anticipated key challenges

Scenario location	Abbreviated case description	Anticipated key challenges
Intensive care unit	77-year-old male patient with pulseless V-tach to PEA arrest	Handoff/sharing of patient’s medical history to arriving cardiac arrest team
Emergency department	67-year-old male patient, ED arrival in PSVT (rhythm with a pulse), decompensates to V-fib arrest, with concerned/loud family member at bedside	• Eliciting key information from family • Managing difficult family member refusing to leave bedside and hindering care team
Pediatric inpatient floor	7-year-old male patient, PEA arrest	• Relatively very rare event • Rapid mobilization of rarely used equipment • Lack of familiarity using the cardiac arrest equipment • Ensuring proper cardiac arrest team arrives (pediatric vs. adult) • Ensuring team response to correct location and awareness of location to reach the pediatric floor (rare arrest location)
Pediatric emergency room	7-year-old male patient, V-fib arrest secondary to respiratory failure	• Relatively rare event • Rapid mobilization of rarely used equipment • Lack of familiarity using the cardiac arrest equipment
Labor & Delivery	34-year-old female, 38 weeks pregnant in early labor stages, previously well, family calls out for help. V-fib arrest	• Relatively very rare event location • Rapid mobilization of rarely used equipment • Lack of familiarity using the cardiac arrest equipment • Ensuring timely activation of both cardiac arrest team plus pediatrics/neonatal teams • Ensuring team response to correct location and awareness of location of L&D to medical team • Rapid escalation to high-risk procedure (resuscitative hysterotomy)
Hospital lobby	62-year-old male, found unresponsive in lobby, V-fib arrest	• Rapid mobilization of equipment to location where there is no cardiac arrest or medical equipment or stretcher • Need for coordinated team response to location and balance of care in lobby vs. expedited transfer to ED
Behavioral health (inpatient)	65-year-old male, V-tach arrest to V-fib arrest	• Relatively rare event location • Restricted access to inpatient psychiatric floor for responding team requiring proactive unlocking of door or staff to meet responding cardiac arrest team for admittance • Ensuring cardiac arrest team able to find location in timely manner • Rapid mobilization of rarely used equipment that is secured in unit • Use of rarely used equipment
Endoscopy suite	65-year-old man, V-fib arrest following anesthetic administration	• Relatively rare event location • Very small physical space requiring coordination and limiting of personnel and equipment into room • Management of crowd control of team members blocking patient access due to confined space • Rapid mobilization of rarely used equipment • Ensuring cardiac arrest team able to find location in timely manner
Cardiac catheterization lab	65-year-old man, STEMI into V-fib arrest	• Cardiac arrest management complicated by physical equipment in room for procedure • Ensuring cardiac arrest team able to find location in timely manner

*V-tach*, ventricular tachycardia. *PEA*, pulseless electrical activity. *PSVT*, paroxysmal supraventricular tachycardia. *V-fib*, ventricular fibrillation. *STEMI*, ST elevation myocardial infarction

### Participants

Participants from a variety of departments were notified at the start of the initiative via monthly emails that there would be unannounced in situ simulations throughout the hospital aimed at evaluating the system response to cardiac arrests. Participants were informed that the simulations would be conducted similar to prior simulations in which they may have participated in the simulation center, similarly comprised of a simulation case followed by structured team debriefing that would be video-recorded. In the introduction that was reinforced prior to the debriefing, pre-briefing information provided included request for confidentiality, fiction contract for simulation and suspension of disbelief, and the objective of the simulation for system assessment and study of team performance and not individual performance. The e-mail-specified participation was voluntary. The number of participants in each in situ simulation varied based on time of day, provider availability to respond, and the unit in which the simulation occurred, all reflecting real-life cardiac arrest team responses. All simulations were facilitated with no fewer than 3 members of the simulation faculty. Debriefings were led by 1–2 clinically and simulation-trained faculty immediately following the scenario as well as 1 member of the simulation team dedicated to recording findings in real-time of the simulation and debriefing. This initiative was approved by the Institutional Review Board of the Icahn School of Medicine at Mount Sinai.

### Approach

Standardized cardiac arrest scenarios were developed by the simulation team in collaboration with stakeholders from each respective department in order to replicate highest fidelity, most common CA scenarios encountered in each area. Each scenario was piloted for clarity, flow, and feasibility to match main objectives and revised. The overarching scenario objectives in all cardiac arrest scenarios throughout the hospital that were the focus of the debriefings included concepts such as organization of team members, leadership, communication, and familiarization and proficiency with equipment and protocols during a cardiac arrest. These were further modified and enhanced based on the unit and location where the in situ simulation occurred.

The simulations utilized equipment appropriate to area of conduction, e.g., Laerdal SimMan 3G (Wappingers Falls, New York) for adult cardiac arrests, Laerdal SimJunior (Wappingers Falls, New York) for pediatric cardiac arrests, and Laerdal SimMom (Wappingers Falls, New York) for obstetric arrests, with accompanying vital sign display monitor. All other equipment utilized during the simulation (e.g., defibrillator, crash carts, intraosseous (IO) drill) was real, from the clinical environment, and obtained by participants in real time during the simulation.

As the simulations occurred in the clinical setting where actual patient care was occurring, the ability to conduct a simulation was balanced with a variety of “no-go” criteria, depending on the unit, under which in situ simulations would be canceled, postponed, moved to another area, or rescheduled [25]. Standard “no-go” criteria were established prior to beginning the study (Additional file 1), were unit specific, and were agreed upon by all major stakeholders (e.g., medical and nursing directors of specific hospital areas).

The simulations were conducted during different shifts and different days of the week to represent a diversity of experiences and maximize capture of LSTs during varied time frames and staffing ensuring more participants would be able to experience and learn from the simulation. The simulations varied in length depending on the unit, objectives, and time for cardiac arrest team response; most were between 5- and 15-min duration. Debriefings following the simulations were conducted for 10–30 min utilizing PEARLS framework [26] of debriefing to capture participant’s reactions, perceived areas of strengths and weaknesses, and summary take-home points, as well as to elucidate LSTs and to instill critical teaching points. PEARLS was utilized to allow participants to reflect on team performance, including perceived system issues and focus on what they, as practicing providers, deem to be major strengths and weakness of cardiac arrest team during case. In addition, components of the PEARLS for Systems’ Integration were used as standardized, and facilitated debriefing points were instilled throughout the session [27]. The scenarios and items in the standardized debriefing discussion points were iteratively updated throughout the initiative in order to emphasize lessons learned that emerged from previous simulations as opportunities for improvement or near misses.

### Outcome measures

The primary outcome measure was the number and type of unique LSTs, and secondary outcomes were key challenges encountered by specific location, LST underlying causes, threat level of individual LSTs, and mitigation steps conducted. LSTs were obtained by simulation-trained staff review of scenario videos coupled with postsimulation debriefing notes from the participants’ and simulation team’s observations and discussion.

Each LST was recorded and categorized into 1 of 4 main categories: equipment, medication, technical skill, or resource/system by 2 independent reviewers, with review and discussion until consensus if disagreement in initial LST category assignment. With a familiarity of previous literature categorizing LSTs [16, 24], an a priori LST mitigation system was established based on the 4 chosen categories to allow for streamlined LST tracking and mitigation. While LST categories demonstrate overlap and many LSTs could be included appropriately into several categories, reviewers categorized each LST to a single, most representative category. Frequencies of each LST were also captured.

Equipment threats were mitigated in collaboration with unit nurse managers, department chairs, and/or materials management/central supply. Technical threats were often directed to department chairs, residency program directors, nurse managers, and nursing education leadership for education. Resource/system threats were directed to hospital operations, nursing leadership, department chairs, and patient safety officers. Mitigation steps were shared with simulation participants in closed loop debriefing so participants could observe LSTs addressed in a timely manner with the goal that participants would be more likely to want to engage in simulation in the future and would recognize the value of taking clinical time away from their day to devote themselves to simulation to improve their clinical environment.

The established Hospital Cardiac Arrest Team Committee as well as the Quality Committee reviewed all unique LSTs to ensure appropriate and timely mitigation as well as reviewed proposed changes to the cardiac arrest protocols and policies based on the reported threats. They further served as a resource should more assistance be needing in mitigating a threat and provided advice if threat mitigation required other hospital resources.

To assign “threat level” of LSTs, all LSTs were reviewed by a physician from the simulation team and a physician from the location of the simulation. These threats were deemed to be imminent, high risk, or non-imminent based on perceived frequency (during simulations as outlined by LST analysis and in real life in that specific unit from experience of physicians from the locations in which the threat occurred) and perceived severity of threat (e.g., to subsequent cardiac arrest patients), each rated on a scale from 1 to 4. A single simulation faculty physician reviewed and rated all LSTs from all cases in conjunction with a discipline-specific faculty physician who reviewed all LSTs for simulations conducted in their area discipline (e.g., one pediatrician rated all pediatric LSTs). Rater training was conducted in advance to review framework for LST identification, categorization, and matrix scoring. No inter-rater reliability was conducted as all ratings were concurrently scored and discussed in real time if discrepancy existed until consensus. All simulations and debriefings were video-recorded using a mounted, hospital-issued tablet, and discussion points and topics were recorded by a scribe. LST identification was made in real time and videos reviewed by experts jointly to ensure accuracy and completeness. Any discrepancies in scores were discussed until consensus was reached. A hazard matrix was used by multiplying the frequency and severity scores [11, 28]. A hazard score of greater than 8 was deemed critical/high risk based on previous literature [11]. Hazard matrix scoring allowed for a framework to prioritize threat mitigation; however, all threats were escalated and mitigated.

In addition to identification of LSTs, mitigation steps and type of required intervention(s) were recorded, and the team utilized closed loop debriefing by which resolution updates were provided back to simulation participants via email. Key challenges by hospital-wide location were delineated to further inform type of mitigating intervention.

## Results

Seventy-four in situ simulations were conducted over the course of 24 months throughout the hospital. Twenty-four in situ simulations occurred on labor and delivery, 23 in the adult ED, 11 in the pediatric ED, 8 in the pediatric floors, 3 in the medical ICU, 2 in behavioral health, 1 in the endoscopy suite, 1 in the cardiac catheterization lab, and 1 in the hospital lobby.

Hundreds of safety threats were identified and analyzed to reduce redundancies yielding 106 unique latent safety threats, a rate of 1.43 unique safety threats per simulation (Table 2).

**Table 2:** Latent safety threat numbers and examples by category

LST type (*N* = 106)	Examples of specific threats
Equipment (21)	Unable to find EZ-IO kit Incorrect needle size in EZ-IO kit Unaware of defibrillator location, delay getting machine Lack of knowledge of how to activate code team within room on L&D Unable to locate laryngeal mask airway and large syringe for its inflation Unable to rapidly obtain scalpel for perimortem C-section Unable to locate step stool
Medication (8)	Team unsure where to locate magnesium Team uncertain on epinephrine dosing in infant Albuterol given via non-rebreather mask while mannequin intubated, so mask left hanging at bedside and albuterol failed to be delivered
Resource/system (41)	Inconsistent response of cardiac arrest team Lack of clear team leader Lack of role designation by leader General role confusion Unclear ideal positioning of rescuers for compressions and airway management Lack of backup system for reaching attending when overhead paging system not heard by attending Overcrowding in clinical space due to redundancy in code team providers responding
Technical skill (36)	Lack of knowledge on how to verify effective ventilations Lack of knowledge on shockable rhythm identification Lack of skill in laryngeal mask airway placement Lack of awareness of need to utilize step stool for compressions Incorrect defibrillator pad placement Incorrect placement of cardiac board Lack of awareness of time to perimortem C-section goal Lack of knowledge about ZOLL defibrillator (e.g., turning it on, utilizing AED mode vs. manual mode, use of CPR feedback mechanism) Failure to provide effective CPR

Twenty-one unique safety threats were identified in the equipment category. Examples of equipment-related LSTs requiring remediation included not being able to locate the IO drill kit, having incorrectly sized needles within the IO kit, and not knowing the location and stocking of various CA equipment. The equipment-related LSTs that occurred most frequently were problems with stepstool use (14), including not being in the room when needed and never found during cardiac arrest and not being able to use or find a backboard (9).

There were 8 unique LSTs within the medication category with the most frequent LST being issues with which medication to give at which time, as well as their respective doses.

Forty-one unique LSTs were identified within the resource/system domain with the most frequent LST noted to be failure to assign roles (27), no identification of a team leader (15), and having an overcrowded environment (12). Other LSTs in this category include not being able to properly activate the cardiac arrest team, failure of the entire cardiac arrest team to arrive, or redundancy in arriving team members causing crowding.

Lastly, 36 unique LSTs were identified within the technical skill category with most common examples being lack of knowledge of or failure to correctly use the defibrillator (29) and lack of knowledge in how to perform effective chest compressions (10).

The team worked to mitigate all 106 unique LSTs with priority mitigation to imminent risk level threats, then high risk threats, followed by non-imminent risk LSTs. Four LSTs were deemed imminent LSTs and deemed to require immediate remediation post debriefing. These included physical safety hazard in the environment (wires on the floor), an incompletely stocked advanced airway cart (missing endotracheal tubes from cart despite inclusion on signed stocking list), bag mask not in designated location and not able to be obtained from alternative location in real time, and no defibrillation pads on the crash cart. Fifteen LSTs had a hazard ratio greater than 8 which was deemed high risk for remediation. Types of intervention included immediate escalation to leadership, hospital-wide cardiac arrest committee review, and/or staff and provider education intervention (Table 3). Depending on the category of threat, a combination of mitigating steps including the immediate fixing of an identified problem, leadership escalation, and programmatic intervention recommendations occurred. As many of the technical skills were deficits in knowledge, program directors were involved in reviewing those LSTs for programmatic education interventions. Similarly, most equipment and resource/system threats were escalated to department and unit leadership for mitigation. An overarching mitigation strategy was incorporation of LSTs that fell into lack of technical skills/knowledge category into standardized debriefing points for all future simulations done in all locations. Examples of specific mitigation strategies include remediation of equipment stocking failures through creation of audit systems for respective equipment.

**Table 3:** Examples of mitigation steps for latent safety threats

Examples of themes/areas for improvement identified	Underlying cause(s) identified	Mitigating action(s) taken	Type of required intervention(s)
Lack of leadership and clear team role delineation	• Inherent variability in team composition by location • Lack of education, training, and practice opportunities • Lack of explicitly clear team composition and expected roles	• Explicit role review at every debriefing and schematic handout creation for ED (in development for other units) • Cardiac arrest team committee revisit of ideal composition of responding cardiac arrest team • Initiation of a cardiac arrest team leader educational curriculum	• Hospital-wide cardiac arrest team committee review • Protocol change • Staff and provider education • Drafting of team member diagram of physical locations by role around the bedside
Chaotic, loud environment during cardiac arrest	• Crowd control not an explicit team role • Variation in cardiac arrest team response; redundancy in responders	• Debriefing with cardiac arrest team leader education and role assignment to include crowd control • Incorporation of nurse manager/supervisor role to address crowd control	• Hospital-wide cardiac arrest team committee review • Protocol change • Staff and provider education
Lack of familiarity with use of defibrillator (including pad placement and modes)	• Lack of education, training, and practice opportunities	• Standardized debriefing teaching points added to simulations to emphasize knowledge of the unit’s defibrillator after every simulation • Simulation center sessions initiated for further defibrillator training and review	• Staff and provider education
Medication delay due to lack of awareness of crash cart stocked medications	• Lack of education, training, and practice opportunities with crash cart	• Simulation center sessions initiated for further hands-on practice/review of crash cart contents, including medications	• Staff and provider education
Delay to pediatric cardiac arrest medication administration	• Low frequency of pediatric cardiac arrests • Lack of education, training, and practice opportunities for application of PALS	• Standardized debriefing teaching points added to pediatric simulations to review PALS rhythms and algorithms regardless of specific simulation case rhythm • Cognitive aid of PALS algorithms to be made available on crash carts	• Hospital-wide cardiac arrest team committee review • Crash carts to be modified to include cognitive aid of PALS algorithms
Insufficient and/or incorrect supply of IO needles stocked in kit^a^	• Stocking error • Lack of “double check”/audit process	• Stocking process reviewed and new audit process added	• -Immediate escalation to leadership • -Protocol change
Delay to cardiac arrest team activation or incorrect activation^a^	• -Lack of education, training, and practice opportunities • “Code blue” labelled button misleading (calls nursing station not cardiac arrest team)	• Standardized debriefing teaching points added to simulations to emphasize knowledge of the unit’s protocol to activate cardiac arrest team • Removal of label “Code Blue” on bedside button; specific education to units with these buttons on how to activate the cardiac arrest team	• Immediate escalation to leadership • Hospital-wide cardiac arrest team committee review • Equipment modification

^a^Critical LST examples based on hazard matrix score > 8. *ED*, emergency department

## Discussion

### The power of in situ simulation

Simulation offers an effective way to test the system for LSTs that impact cardiac arrest resuscitation and safety in the actual clinical environment. This initiative revealed deficiencies in knowledge of cardiac arrest team roles, medication management in real time, and equipment knowledge, all skills that can be practiced through simulation. In situ simulation allows for discovery of areas of weakness of an interdisciplinary group of cardiac arrest providers and can capture deficiencies in teamwork, leadership, and team roles, as well as communication skills.

Since many responding cardiac arrest teams form ad hoc, it is imperative that team members have the opportunity to practice together. One in situ simulation on labor and delivery, for example, revealed effective compressions, teamwork, and knowledge of ACLS; however, the team was unable to rapidly obtain a scalpel for resuscitative hysterotomy due to a lack of standardization of scalpel location outside the operating room with no team member ever previously needing to emergently locate a scalpel.

By completing these simulations unannounced, it further allows for the evaluation of real-time threats from earliest case stages (e.g., team successfully finding location of the cardiac arrest patient based on operator page message and team member arrival and need for leader identification and crowd management). From a systems’ perspective, it eliminates some of the artifacts natural to a simulation center or announced in situ simulation such as the natural tendency to prepare for upcoming case or, more simply, physically being aware of location of simulation ahead of time. In a system with variability in team members responding based on schedule and staffing, it is nearly impossible for full complement of the cardiac arrest team to practice together or potentially all members to be aware of team composition ahead of time. It allows them in their clinical environment to come together as a team (with many coming together for the first time), identify themselves and their roles, and perform cardiac arrest resuscitation using authentic equipment in the real-life confines of the space they practice with opportunities for debriefing, question and answer, and feedback afterwards. In addition, throughout the simulations, as with real-life cardiac arrest responses, new cardiac arrest team members often continued to arrive at staggered times representing the reality of ever-changing conditions and team compositions and forcing alterations from team members’ original roles. While a variety of cardiac arrest courses may use simulation as a teaching tool for the medicine and steps of an algorithm, the opportunity for a complete, multidisciplinary team consisting of the same members that would respond if a cardiac arrest occurred on that floor at that time to adapt, transfer leadership, and perform new tasks, with real equipment in the clinical arena in which they regularly work, and then debrief together is paramount for cardiac arrest resuscitation assessment and strengthening from a team and systems’ perspective. Through this initiative, LSTs in each of these steps have been observed because the simulations occurred in situ.

While there is an opportunity cost for conducting unannounced, in situ simulations such as taking people and resources away from clinical time, replacing authentic equipment, and resetting the area for clinical use, in this study, LSTs were able to be identified and mitigated in each of the above steps because the simulations occurred both unannounced and in situ.

### Mitigating LSTs for the present and future

In situ simulation does more than offer a way to identify LSTs. It offers a unique ability to perform systems testing to mitigate potential threats. One simulation of an adult cardiac arrest identified an LST regarding the relatively new initiation of IO drill kits in that location. The resident physician asked a nurse for the IO kit, and the nurse was correctly and quickly able to retrieve the new kit on the unit. The resident, however, realized that the kit contained only pediatric-sized needles and therefore could not place an IO line in the adult cardiac arrest patient. That equipment LST was discussed during the debriefing and then addressed immediately by the simulation team with the team responsible for stocking and checking the IO kits. Coincidentally, a real cardiac arrest occurred on that exact unit later in the day during which the staff was able to utilize the newly, and correctly, stocked IO kit to save a real life. This particular example not only underscores the value of completing simulation in situ if the main goal is to address LSTs from a systems’ approach, but it also emphasizes the importance of maintaining a system for addressing and mitigating unmasked LSTs and closing the loop, often in real time based on urgency of LST identified. While that 1 kit happened to have been identified as having the wrong needles during that 1 particular simulation and was fixed by the simulation team in real time, it is important to recognize from a systems’ perspective that other kits could have also been stocked incorrectly. Because of the streamlined approach to threat mitigation, soon after, the team was able to work with hospital leadership and materials management to create a new audit system for the kits hospital wide.

This type of hospital-wide mechanism of reporting and addressing LSTs was crucial for the success of the initiative. Mitigation steps were shared with simulation participants to ensure closed loop debriefing so participants could observe LSTs addressed in a timely manner with the goal that participants would be more likely to want to engage in simulation in the future and would recognize the value of taking clinical time away from their day to devote themselves to simulation to improve their clinical environment.

### Using in situ simulation as a needs assessment

This initiative identified numerous LSTs in all 4 domains. As a result of the simulations, standardized debriefing points were iteratively expanded for all future cardiac arrest simulations, tailored to the respective units. For example, lack of knowledge and ability to operate the defibrillator was noted in 29 instances. As a result, defibrillator education is conducted post simulation on all units regardless of simulation performance in order to ensure all learners are proficient in its use. In the labor and delivery unit, cognitive aid signs were hung prompting providers to perform left lateral uterine displacement. In addition, reviewing the goal time of 4 min to initiate cesarean section is reviewed every debriefing, even if it was completed appropriately, due to the many simulations during which it was not. This example of iterative expansion of debriefing pearls was conducted to ensure all members of the team are aware of the ACLS modification in obstetrics. Similar application of findings was utilized throughout other departments including reviewing the Broselow™ tape in pediatrics.

This initiative has also led to the formation of a team leader simulation curriculum for the cardiac arrest team leaders (targeting senior medicine residents, the cardiac arrest team leaders at this institution) based on the leadership and communication failures identified. The LSTs revealed numerous instances of failure to identify a team leader, poor communication with minimal to no closed loop communication, poor transition of leadership from the initial team to the responding cardiac arrest team, failure to assign roles, and failure to have adequate crowd control. The new curriculum is aimed at educating these providers not only in the ACLS and PALS algorithms but also on specific skills such as communication, fostering situational awareness, overall leadership, and command of a chaotic environment.

### Limitations

This was a single-center study, and varying numbers of simulations were conducted by area with most robust inclusion of the adult ED and labor and delivery, with far lower numbers of simulations in certain areas such as just 1 simulation to date in endoscopy and cardiac catheterization suite. Lack of more distributed representation may limit ability to generalize to hospital-wide cardiac arrest team performance; however, all adult areas (outside adult ED and ICU) are unified by same cardiac arrest team coverage. Future increased frequency of simulations could be conducted in additional areas to continue to utilize in situ simulation to educate and improve safety.

While there are many different systems and ways to categorize LSTs [16], it is merely a construct to aid in analysis and discussion around captured LSTs. This initiative assigned each LST to a single, best-fit category. There may be significant overlap among domains with certain LSTs, but regardless of where they were consistently coded, the focus was on capturing and mitigating, regardless of categorization.

### Future direction/next steps

This large-scale cardiac arrest team initiative paves the way for further future expansion and rollout throughout other areas of the hospital and, potentially, other hospital centers. Creation, implementation, and formalization of this initiative and system for capturing and mitigating LSTs as a training and safety enhancement tool may be expanded in the future to encompass use in other hospitals and practice setting types, such as with trauma teams and stroke teams.

## Conclusion

This paper reports on the use of hospital-wide in situ cardiac arrest simulation to both identify and mitigate LSTs. In situ simulation offers a unique perspective on systems’ assessment to identify potential risks to patient safety in real time by allowing providers to simulate with their own teams utilizing real equipment in their real practice settings. This initiative further describes the use of a system that was created in which LSTs could be escalated urgently, mitigated, and resolutions conveyed back to participants to provide closed loop debriefing. Lastly, this hospital-wide, multidisciplinary initiative additionally served as an educational needs assessment allowing for informed, iterative education and systems improvement initiatives targeted to areas of LSTs and areas of opportunity.

## Supplementary Information

**Additional file 1.**

## Data Availability

The datasets used and/or analyzed during the current study are available from the corresponding author on reasonable request.

## Abbreviations

LST: Latent safety threat
ED: Emergency department
ICU: Intensive care unit
ACLS: Advanced cardiac life support
PALS: Pediatric advanced life support
BLS: Basic life support
