# Supplementary material for: Hospital-wide cardiac arrest in situ simulation to identify and mitigate latent safety threats
Source: Adv Simul (Lond). 2022 May 21;7:15. doi: 10.1186/s41077-022-00209-0 (PMC9124397; doi:10.1186/s41077-022-00209-0)
Supplement: Supplementary file 1 — Additional file 1. [file 41077_2022_209_MOESM1_ESM.docx]

Appendix A

**NO GO CRITERIA**

List is not exhaustive and ultimate No Go decision at discretion of Nursing leadership/Unit Nurse Manager and/or Attending/Chief Resident Physician.

Goal: varied timeframes to ensure staff exposure; will limit simulations on Mondays per Nursing Leadership.

Before event: sim staff to confirm “Go ahead” from Unit Nurse Manager in selected in situ location.

·         Active high acuity or decompensating patient on proposed floor/unit area

·         High acuity patient or decompensating patient care concluding within last 2 hours since proposed simulation

·         Active cardiac arrest or decompensating patient in nearby patient care area requiring reallocation of nursing and staff to assist

·         Attending and/or charge nurse discretion​
